# Atividade Física no Tempo Livre e Incidência de Hipertensão Arterial em Participantes do ELSA-Brasil

**DOI:** 10.36660/abc.20230734

**Published:** 2024-07-15

**Authors:** Tarcísio C. Souza, Sheila M. A. Matos, Maria da C. C. de Almeida, Maria J. M. Fonseca, Maria del Carmen B. Molina, Rosane H. Griep, Cristiano P. S. Pitanga, Francisco J. G. Pitanga

**Affiliations:** 1 Universidade Estadual do Sudoeste da Bahia Jequié BA Brasil Programa de Pós-Graduação em Educação Física (PPGEF) – Universidade Estadual do Sudoeste da Bahia (UESB), Jequié, BA – Brasil; 2 Universidade Federal da Bahia Instituto de Saúde Coletiva Salvador BA Brasil Instituto de Saúde Coletiva (ISC) – Universidade Federal da Bahia (UFBA), Salvador, BA – Brasil; 3 Fundação Oswaldo Cruz Instituto Gonçalo Moniz Salvador BA Brasil Instituto Gonçalo Moniz (IGM) – Fundação Oswaldo Cruz (FIOCRUZ), Salvador, BA – Brasil; 4 Fundação Oswaldo Cruz Escola de Saúde Pública Rio de Janeiro RJ Brasil Escola de Saúde Pública – Fundação Oswaldo Cruz (FIOCRUZ), Rio de Janeiro, RJ – Brasil; 5 Universidade Federal do Espirito Santo Vitória ES Brasil Programa de Pós-graduação em Saúde Coletiva (PPGSC) – Universidade Federal do Espirito Santo (UFES), Vitória, ES – Brasil; 6 Laboratório de Educação em Ambiente e Saúde Fundação Oswaldo Cruz Rio de Janeiro RJ Brasil Laboratório de Educação em Ambiente e Saúde – Fundação Oswaldo Cruz (FIOCRUZ), Rio de Janeiro, RJ – Brasil; 7 Universidade Católica do Salvador Salvador BA Brasil Universidade Católica do Salvador(UCSAL), Salvador, BA – Brasil; 8 Universidade Federal da Bahia Salvador BA Brasil Programa de Pós-graduação em Ciências da Reabilitação (PPG-REAB) - Universidade Federal da Bahia (UFBA), Salvador, BA – Brasil

**Keywords:** Doenças Cardiovasculares, Epidemiologia, Estudos Longitudinais, Saúde Pública

## Abstract

**Fundamento:**

Evidências apontam que a atividade física (AF) apresenta efeito protetor para as doenças crônicas, incluindo a hipertensão arterial (HA).

**Objetivo:**

Este estudo investigou, de forma longitudinal, a associação entre as mudanças na atividade física no tempo livre (AFTL) e a incidência de HA em participantes do ELSA-Brasil.

**Métodos:**

Foram analisados dados de 8.968 participantes em dois momentos distintos (2008-2010 e 2012-2014). Foi utilizado o Questionário Internacional de Atividade Física (IPAQ), versão longa, para avaliação da AFTL. A associação entre AFTL e HA foi testada por regressão de Poisson com estimativa do risco relativo (RR), com nível de significância de 5% e intervalo de confiança de 95%.

**Resultados:**

Quando a variável nível de AFTL foi categorizada em suficiente e insuficiente, não foram encontradas associações estatisticamente significantes entre AFTL e a incidência HA em função das mudanças na AF durante o seguimento. No entanto, a variável AFTL quando categorizada em inativo, pouco ativo, ativo e muito ativo, observou-se associação estatisticamente significante entre AFTL e HA em participantes classificados como muito ativos fisicamente. O risco de HA foi reduzido em 35% entre homens RR 0,65 (IC 95% 0,50-0,86) e em 66% entre as mulheres RR 0,34 (IC 95% 0,20-0,58) que mantiveram altos níveis de AFTL em ambos os momentos do seguimento.

**Conclusão:**

Esses resultados sugerem que a manutenção de altos níveis de AF ao longo do tempo está associada a um menor risco de desenvolver HA, destacando a importância da AF na prevenção dessa condição, tanto para homens quanto para mulheres.

**Figure:**
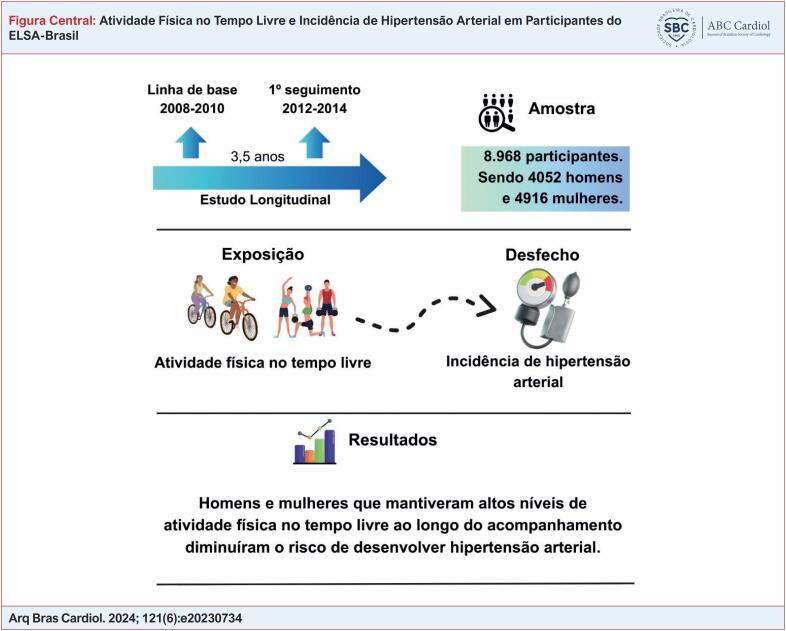

## Introdução

As doenças cardiovasculares destacam-se como a principal causa de óbitos no mundo.^1 - 3^ Os países latino-americanos detêm altas taxas de mortalidade por doenças cardiovasculares, sendo o Brasil o país com as maiores taxas.^2^ A hipertensão arterial (HA), por sua vez, figura como a mais comum entre as doenças cardiovasculares, e o principal fator de risco para as doenças cerebrovasculares. ^3 , 4^

A HA frequentemente se desenvolve devido a uma interação complexa de fatores de risco associados ao estilo de vida, predisposição genética e envelhecimento. Essa condição afeta todas as faixas socioeconômicas de ambos os sexos, mas apresenta as taxas mais elevadas entre os homens, especialmente em nações de renda média e baixa.^4^

A Sociedade Brasileira de Cardiologia aponta em sua última diretriz de HA que a realização consistente de atividade física (AF) desempenha um papel crucial na prevenção da HA, além de contribuir para a redução da pressão arterial (PA) em pessoas que já apresentam essa condição. Esse efeito benéfico pode ser atribuído tanto a mudanças diretas nos aspectos hemodinâmicos quanto a modificações indiretas que ocorrem por meio de adaptações nutricionais, metabólicas e comportamentais.^1 , 2 , 4^

Estudos desenvolvidos com dados do ELSA-Brasil.^5 , 6^ indicam associação inversa entre atividade física no tempo livre AFTL e HA, bem como com eventos cardiovasculares, porém foram realizados de forma transversal.

A partir desse cenário, o presente estudo poderá adicionar conhecimentos ao tema, principalmente na perspectiva de um melhor entendimento sobre as possíveis mudanças nos padrões da AFTL durante o acompanhamento da população estudada e a incidência de HA. Além disso, poderá fomentar a formulação de políticas públicas que promovam e incentivem a prática de AFTL por um número, cada vez, maior de pessoas.

Sendo assim, este estudo tem como objetivo investigar, de forma longitudinal, a associação entre a AFTL e a incidência de HA em participantes do ELSA-Brasil.

## Métodos

### População e amostra

O ELSA-Brasil é um estudo de coorte com 15.105 servidores públicos ativos e aposentados, 35 a 74 anos, de seis instituições de nível superior. O principal objetivo é investigar a incidência e a progressão de diabetes e doenças cardiovasculares, inclusive a HA, e seus fatores associados, cujos detalhes metodológicos foram descritos previamente.^7 , 8^ Para o presente estudo foram selecionados os participantes da linha de base (2008-2010), acompanhados até o 1º seguimento (2012-2014).

Para a composição da amostra deste estudo, foram selecionados exclusivamente indivíduos com PA normal (normotensos), excluindo-se qualquer pessoa com diagnóstico prévio de hipertensão (hipertensos) ou que estivesse em uso de medicamentos para o tratamento da HA. Todos os participantes responderam os questionários sobre AF e com dados completos relativos às variáveis envolvidas na análise, totalizando 8968 participantes, sendo 4916 mulheres e 4052 homens.

O ELSA-Brasil foi aprovado pela Comissão Nacional de Ética em Pesquisa (CONEP) e em todos os Comitês de Ética em Pesquisa dos seis centros de investigações envolvidos. Todos os participantes assinaram o termo de consentimento livre e esclarecido (TCLE), sendo garantidos o sigilo e a confidencialidade dos dados.

### Produção de dados

Os dados foram coletados por uma equipe de entrevistadores e aferidores treinados e certificados por um comitê de controle de qualidade,^7^ capacitados a executar o protocolo do estudo em qualquer Centro de Investigação ELSA-Brasil. Foram realizadas entrevistas presenciais na aplicação dos blocos de questionários para obtenção das informações sobre idade, raça/cor escolaridade, renda familiar, tabagismo e consumo de sal. Além disso, foram coletadas informações sobre medidas antropométricas de peso, estatura e circunferência da cintura. O peso corporal foi obtido pela manhã, após 8 a 12 horas de jejum e com o participante sem sapatos e com roupas leves. Foi usada balança eletrônica Toledo^®^, com capacidade para até 200kg. Para medida da estatura, foi usado estadiômetro da marca SECA^®^, com o participante posicionado de pé e seguindo rigorosamente o plano de Frankfurt. A obesidade foi identificada por meio do índice de massa corporal (IMC), aplicando-se a equação IMC = peso (kg)/altura(m)^2^.

### Avaliação da atividade física

Para identificação e quantificação da AF, foi utilizado o módulo de AFTL do International Physical Activity Questionnary (IPAQ), versão longa, que é constituído de questões relativas à frequência e à duração de atividades físicas (caminhada, AF moderada e AF vigorosa) desenvolvidas no trabalho, no deslocamento, nas atividades domésticas e no tempo livre.^9^ A AF foi mensurada em minutos/semana por meio da multiplicação da frequência semanal pela duração de cada uma das atividades realizadas. Para efeito desse estudo, utilizou-se a AFTL, com a seguinte categorização: 0 = inativo fisicamente (menos de 10 minutos de qualquer AF por semana); 1 = pouco ativo (entre 10 minutos e menos de 150 minutos de caminhada/moderada por semana, ou entre 10 minutos e menos de 60 minutos de atividades vigorosas por semana, ou entre 10 minutos e menos de 150 minutos por semana de qualquer combinação de caminhada moderada e vigorosa); 2 = fisicamente ativo (pelo menos 150 minutos de caminhada/atividade moderada por semana, ou pelo menos 60 minutos de atividades vigorosas por semana, ou pelo menos 150 minutos por semana de qualquer combinação de AF moderada e vigorosa); 3 = muito ativo (pelo menos 150 minutos de atividades vigorosas por semana, ou pelo menos 60 minutos de atividades vigorosas por semana mais 150 minutos por semana de qualquer combinação de AF moderada e vigorosa).

Para as análises com a AFTL categorizada em dois grupos, foram considerados como insuficientemente ativos aqueles classificados como pouco ativos, e como suficientemente ativos aqueles classificados como ativos. Para as análises com a AFTL com quatro categorias, foram considerados os inativos, pouco ativos, ativos e muito ativos.

### Avaliação da hipertensão arterial

A HA foi definida como PA sistólica ≥ 140 mm/Hg e PA diastólica ≥ 90 mm/Hg. A PA foi aferida com esfigmomanômetro digital (Omron HEM-705CP). As medidas de PA foram realizadas após cinco minutos de repouso, com o participante de bexiga vazia, sentado ereto, com as costas relaxadas e apoiadas no encosto, pés apoiados, pernas descruzadas e braço esquerdo apoiado na altura do coração. Foram realizadas três aferições com intervalos de um minuto. A PA utilizada no presente estudo foi estabelecida pela média aritmética da segunda e terceira medida.

### Covariáveis

Para as covariáveis, adotou-se a seguinte categorização: Sexo: homem e mulher; Para raça/cor: pretos, pardos, brancos, asiáticos, indígenas; Para a idade: entre 34 e 50 anos, entre 51 e 60 anos e >60 anos; Para escolaridade, foram estabelecidas quatro categorias: fundamental incompleto, fundamental completo, médio completo e superior completo/pós-graduação. A renda familiar foi categorizada em: até 2 salários mínimos, de 2 até 8 salários mínimos, de 8 até 18 salários mínimos e acima de 18 salários mínimos. O tabagismo atual foi categorizado em não e sim; Para o IMC: < 30 kg/m², = ≥ 30 kg/m²; Para o consumo de sal/dia: < 5g e = ≥ 5g.

### Análise dos dados

As medidas descritivas (proporções) foram calculadas para todas as variáveis categorizadas. As análises foram estratificadas por sexo *a priori* e comparadas por meio do teste Qui-quadrado. As associações foram analisadas por meio de regressão de Poisson, estimando-se o Risco Relativo (RR) com intervalo de confiança a 95%. Foram consideradas como potenciais confundidoras ou modificadoras de efeito as seguintes variáveis: idade, IMC, tabagismo, renda familiar, consumo de sal, raça/cor e escolaridade.

A verificação da modificação do efeito foi feita por meio de estratificação com a observação das medidas pontuais estrato-específicas e os seus intervalos e confiança. Quando a medida pontual de um fator, em determinado estrato específico, não estava contida no intervalo de confiança do outro fator no mesmo estrato, isso indicava modificação de efeito. A análise das possíveis variáveis de confundimento foi realizada pelo procedimento backward, por meio de regressão de Poisson. A análise começando com o modelo completo, seguido pela remoção um a um dos potenciais confundidores. Quando observada modificação igual ou superior a 10% na associação pontual entre AFTL e HA, a variável é considerada confundidora.^10^ Apesar de que no processo de modelagem não foram identificadas variáveis modificadoras de efeito e/ou confundidoras, optou-se pelo ajuste por idade, uma vez que, teoricamente, tanto a AFTL quanto HA se relacionam com idade.

Foram feitas análises longitudinais, calculando o RR entre AFTL e HA tendo como referência a categoria insuficientemente ativo para a análise com duas categorias, e o grupo inativo como referência para a análise com quatro categorias.

Foi utilizado o teste de Mantel-Haenszel para testar a homogeneidade dos valores do RR entre os estratos de cada variável. O nível de significância adotado na análise estatística foi de 5%. O intervalo de confiança foi estabelecido em 95%. Empregou-se o programa estatístico STATA^®^ versão 17.0

## Resultados

Um total de 4.052 homens e 4.916 mulheres foi incluído na análise. As características da amostra estão apresentadas na Tabela 1 . A incidência de HA no primeiro seguimento foi de 16,9%, sendo 12,8% entre as mulheres e de 21,9% entre os homens, todavia a maior parte dos participantes era do sexo feminino (55%), dentre elas 37,8% tinham idade entre 34-50 anos. Observa-se também que as mulheres têm maior renda familiar e são mais ativas no tempo livre, enquanto os homens são mais tabagistas, mais hipertensos e apresentam maior proporção de participantes muito ativos no tempo livre. Observa-se ainda que não existem diferenças estatisticamente significantes entre homens e mulheres com relação à idade. Cerca de 16% de ambos os sexos eram suficientemente ativos na linha de base e se tornaram insuficientemente ativos, e aproximadamente 13% dos insuficientemente ativos se tornaram suficientemente ativos. Cerca de 8% permaneceram muito ativos fisicamente nos dois momentos do acompanhamento.

**Tabela 1:** Distribuição dos participantes segundo características selecionadas e sexo. ELSA-Brasil 2008-2014

		Homens	Mulheres	p-valor
Idade (anos) - n (%)		(4.052)	(4.916)	
	34-50	1642 (46,71)	1859 (53,10)	
	51-60	1427 (44,32)	1793 (55,68)	
	> 60	983 (43,75)	1264 (56,38)	0.030
Raça/cor autoreferrida - n (%)				
	Pretos	479 (41,73)	680 (58,67)	
	Pardos	1229 (49,62)	1248 (50,38)	
	Brancos	2171 (44,05)	2758 (55,95)	
	Asiáticos	68 (30,49)	155 (69,51)	
	Indígenas	50 (60,24)	33 (39,76)	0,00
Renda familiar (salário mínimo) - n (%)				
	Até 2	40 (42,55)	54 (57,45)	
	De 2 até 8	1559 (47,48)	1715 (52,22)	
	De 8 até 18	1385 (40,82)	2008 (59,18)	
	Acima de 18	1041 (48,13)	1922 (51,87)	0,00
Escolaridade - n (%)				
	Fundamental incompleto	253 (71,67)	100 (28,33)	
	Fundamental completo	303 (60,84)	195 (39,16)	
	Médio completo	1323 (45,06)	1613 (54,94)	
	Superior completo/Pós-graduação	2173 (41,94)	1756 (49,84)	0,00
Tabagismo atual - n (%)				
	Não	2276 (41,90)	3156 (58,10)	
	Sim	1767 (50,71)	1756 (49,84)	0,00
IMC - n (%)				
	< 30 kg/m²	3354 (46,81)	3811 (53,19)	
	≥ 30 kg/m²	698 (38,71)	1015 (61,19)	0,00
Consumo de sal - n (%)				
	< 5g	203 (22,61)	695 (77,39)	
	≥ 5g	3849 (47,70)	4121 (52,30)	0,00
Mudanças na AFTL (entre as etapas do estudo- n (%)				
	AFTL insuficiente - AFTL insuficiente	1296 (39,12)	2017 (60,88)	
	AFTL suficiente - AFTL suficiente	217 (45,30)	262 (54,70)	
	AFTL suficiente - AFTL insuficiente	637 (44,73)	787 (55,27)	
	AFTL insuficiente - AFTL suficiente	514 (45,65)	612 (54,35)	0,00
Níveis de AFTL - n (%)				
	Inativo	888 (38,81)	1400 (61,19)	
	Pouco ativo	217 (45,30)	262 (54,70)	
	Ativo	411 (46,97)	464 (53,03)	
	Muito ativo	461 (63,24)	268 (36,24)	0,00
Hipertensão arterial - n (%)				
	Não	3148 (42,45)	4267 (57,55)	
	Sim	887 (58,43)	631 (41,57)	0,00

* Os valores para homens e mulheres foram comparados por meio do teste qui-quadrado. As somas dos estratos nem sempre serão iguais, devido a perda de informações de algumas variáveis. IMC: índice de massa corporal; AFTL: atividade física no tempo livre.

Como observado na tabela 2 , não houve associações estatísticas significantes entre mudanças na AFTL e HA entre os participantes quando utilizamos a classificação em duas categorias.

**Tabela 2:** Associação entre mudanças da AFTL entre dois seguimentos e incidência de HA em participantes do ELSA-Brasil (sexo masculino e feminino)

Mudanças na AFTL	HomensRR (IC 95%)	MulheresRR (IC 95%)
AFTL insuficiente – AFTL insuficiente	1,00	1,00
AFTL suficiente – AFTL suficiente	1,00 (0,75-1,35)	1,06 (0,76-1,48)
AFTL insuficiente – AFTL suficiente	0,95 (0,78-1,16)	0,87 (0,69-1,10)
AFTL suficiente – AFTL insuficiente	0,81 (0,64-1,02)	0,80 (0,62-1,04)

Análise ajustada por idade. RR: risco relativo; AFTL: atividade física no tempo livre.

A tabela 3 apresenta a análise utilizando o nível de AFTL em quatro categorias, a qual indicou associação estatística entre AFTL e HA em participantes muito ativos: risco de HA reduzido em 35% entre homens RR = 0,65 (IC 95% 0,50-0,86) e risco de HA reduzido em 66% entre as mulheres RR = 0,34 (IC 95% 0,20-0,58). A figura central resume as principais informações do manuscrito.

**Tabela 3:** Associação entre níveis de AFTL e incidência de HA em participantes do ELSA-Brasil (sexo masculino e feminino)

Níveis de ATFL	Homens RR (IC 95%)	Mulheres RR (IC 95%)
Inativos – Inativos	1,00	1,00
Pouco Ativos – Pouco ativos	1,03 (0,76-1,40)	0,99 (0,71-1,39)
Ativos – Ativos	0,83 (0,64-1.07)	0,77 (0,58-1,03)
Muito Ativos – Muito ativos	0,65 (0,50-0,86)	0,34 (0,20-0,58)

Análise ajustada por idade. RR: risco relativo; AFTL: atividade física no tempo livre.

## Discussão

Este estudo analisou associações entre mudanças da AFTL durante o período de acompanhamento e incidência de HA. A AFTL, particularmente no grupo muito ativo fisicamente, esteve associada a um menor risco de desenvolver HA ao longo do tempo.

Após análise estatística, considerando os participantes classificados em duas categorias, os primeiros resultados não revelaram associações estatísticas significantes entre AFTL e HA. Por outro lado, entre os participantes classificados em quatro categorias, aqueles considerados como "muito ativos" fisicamente, tanto homens quanto mulheres que mantiveram altos níveis de AFTL ao longo do estudo apresentaram um risco significativamente menor de desenvolver HA. Além disso, as mulheres tiveram uma redução ainda mais acentuada no risco em comparação aos homens.

O risco significativamente menor de desenvolver a HA em indivíduos muito ativos pode ser explicada por vários fatores. Primeiramente, a intensidade e a regularidade da AF desempenham um papel crucial na redução da PA.^11^ Pessoas muito ativas geralmente se envolvem em AF mais intensas e frequentes, o que contribui para melhorar a saúde cardiovascular e a capacidade do corpo de regular a PA de forma mais eficaz.^11 , 12^

Além disso, a AF intensa pode levar a modificações benéficas em outros fatores de risco associados à hipertensão, como o controle do peso, a sensibilidade à insulina e a função vascular. Pessoas muito ativas gastam mais energia mantendo-se com menor percentual de gordura corporal, o que pode estar ligado a uma menor incidência de HA.^2 , 3 , 11^

Também é importante considerar que a AF intensa geralmente está associada a estilos de vida mais saudáveis em termos de dieta e hábitos comportamentais. Indivíduos muito ativos muitas vezes são mais conscientes de sua saúde, o que pode levar a escolhas alimentares melhores e redução de fatores de risco, como o consumo de sal.^3 , 11 , 12^

No entanto, isso não significa que a AF moderada seja ineficaz na prevenção da HA, conforme sugeriu estudo,^13^ que para prevenção de condições como hipertensão, eventos cardiovasculares e até diabetes, a realização de AFTL com intensidade moderada a vigorosa já se mostrou eficiente.

Nessa perspectiva, um estudo transversal prévio com dados da linha de base do ELSA-Brasil, mostrou associação inversa entre AFTL e HA nos indivíduos que praticavam AFTL com intensidade moderada.^5^ Na mesma linha, outro estudo apontou que a AF no domínio doméstico, de baixa e moderada intensidade foi associada com um menor risco para HA.^14^

Por outro lado, um estudo também com dados do ELSA-Brasil, verificou a associação entre AFTL e AF no deslocamento com escores de risco cardiovascular, mostrando que apenas a AFTL com duração e intensidade mais elevados esteve inversamente associada com HA e eventos cardiovasculares.^6^

Nossos resultados corroboram outros estudos longitudinais, metanálises e até ensaios clínicos,^11 , 15 , 16^ os quais demonstram que a redução no risco para HA se atrela de forma progressiva ao volume e a intensidade da AF praticada, e que quanto maior o volume e/ou intensidade da AF, menor será o risco para o desenvolvimento de HA.

É fundamental enfatizar que a nossa pesquisa tem algumas características notáveis, apesar da população estudada consistir em uma coorte de servidores públicos voluntários. Primeiramente, a coorte é composta por um número substancial de participantes de seis capitais brasileiras. Essa diversidade regional é um aspecto positivo do estudo, pois pode proporcionar percepções sobre a HA em diferentes contextos geográficos e culturais do Brasil. Isso, por sua vez, contribui para a generalização dos resultados e a compreensão mais abrangente dos fatores que afetam a hipertensão na população brasileira.

Além disso, a escolha de servidores públicos voluntários como grupo de estudo pode apresentar vantagens, uma vez que esse grupo pode ter um melhor acesso a cuidados de saúde, informações sobre saúde e consciência sobre a importância de monitorar e controlar a PA. Portanto, a pesquisa pode fornecer informações valiosas sobre estratégias de prevenção e conscientização que podem ser aplicadas em um contexto mais amplo.

Embora a população do estudo possa não ser totalmente representativa da população em geral, suas características únicas podem fornecer compreensões valiosas sobre a HA e servir como base para futuras pesquisas e intervenções direcionadas à saúde cardiovascular no Brasil.

Um aspecto desfavorável foi o uso de questionários, já que as respostas podem ser imprecisas devido à dependência da memória. Sugerimos adotar o acelerômetro para medições mais objetivas da AF em futuros estudos, como já está sendo feito no ELSA-Brasil.

## Conclusão

De acordo com os resultados deste estudo, a AFTL se associou à redução do risco de HA em participantes que mantiveram níveis mais altos de AFTL em ambos os momentos analisados. Quanto mais ativos fisicamente, menor o risco de desenvolver HA, independentemente do sexo. Isso destaca o papel crucial da AF na redução do risco de hipertensão, especialmente quando praticada de forma consistente. Estes achados sublinham a importância de promover um estilo de vida ativo como medida preventiva contra a HA.

Futuras pesquisas devem continuar a explorar a associação entre AFTL mais vigorosa e HA, incluindo o uso de acelerometria, para fornecer diretrizes mais sólidas para políticas públicas de promoção da AF e saúde.
