# Supplementary material for: Atividade Física no Tempo Livre e Incidência de Hipertensão Arterial em Participantes do ELSA-Brasil
Source: Arq Bras Cardiol. 2024 Jul 15;121(6):e20230734. [Article in Portuguese] doi: 10.36660/abc.20230734 (PMC12092042; doi:10.36660/abc.20230734)
Supplement: Translation

# Translation: Leisure Time Physical Activity and Incidence of Arterial Hypertension in ELSA-Brasil Participants

**DOI:** 10.36660/abc.20230734i

**Authors:** Tarcísio C. Souza, Sheila M. A. Matos, Maria da C. C. de Almeida, Maria J. M. Fonseca, Maria del Carmen B. Molina, Rosane H. Griep, Cristiano P. S. Pitanga, Francisco J. G. Pitanga

**Affiliations:** 1 Jequié BA Brazil Programa de Pós-Graduação em Educação Física (PPGEF) – Universidade Estadual do Sudoeste da Bahia (UESB), Jequié, BA – Brazil; 2 Salvador BA Brazil Instituto de Saúde Coletiva (ISC) – Universidade Federal da Bahia (UFBA), Salvador, BA – Brazil; 3 Salvador BA Brazil Instituto Gonçalo Moniz (IGM) – Fundação Oswaldo Cruz (FIOCRUZ), Salvador, BA – Brazil; 4 Rio de Janeiro RJ Brazil Escola de Saúde Pública – Fundação Oswaldo Cruz (FIOCRUZ), Rio de Janeiro, RJ – Brazil; 5 Vitória ES Brazil Programa de Pós-graduação em Saúde Coletiva (PPGSC) – Universidade Federal do Espirito Santo (UFES), Vitória, ES – Brazil; 6 Rio de Janeiro RJ Brazil Laboratório de Educação em Ambiente e Saúde – Fundação Oswaldo Cruz (FIOCRUZ), Rio de Janeiro, RJ – Brazil; 7 Salvador BA Brazil Universidade Católica do Salvador (UCSAL), Salvador, BA – Brazil; 8 Salvador BA Brazil Programa de Pós-graduação em Ciências da Reabilitação (PPG-REAB) - Universidade Federal da Bahia (UFBA), Salvador, BA – Brazil

**Keywords:** Cardiovascular Diseases, Epidemiology, Longitudinal Studies, Public Health

## Abstract

**Background:**

Evidence indicates that physical activity (PA) has a protective effect against chronic diseases, including high arterial hypertension (AH).

**Objective:**

This study investigated, longitudinally, the association between changes in leisure time physical activity (LTPA) and the incidence of hypertension in ELSA-Brasil participants.

**Methods:**

Data from 8,968 participants were analyzed at two different times (2008-2010 and 2012-2014). The International Physical Activity Questionnaire (IPAQ), long version, was used to assess LTPA. The association between LTPA and AH was tested using Poisson regression with relative risk (RR) estimation, with a significance level of 5% and a 95% confidence interval.

**Results:**

When the LTPA level variable was categorized as sufficient and insufficient, no statistically significant associations were found between LTPA and AH incidence as a function of changes in PA during follow-up. However, when the LTPA variable was categorized as inactive, little active, active, and very active, a statistically significant association was observed between LTPA and AH in participants classified as very physically active. The risk of AH was reduced by 35% among men RR 0.65 (95% CI 0.50-0.86) and by 66% among women RR 0.34 (95% CI 0.20-0.58) who maintained high levels of LTPA at both moments of follow-up.

**Conclusion:**

These results suggest that maintaining high levels of PA over time is associated with a lower risk of developing AH, highlighting the importance of PA in preventing this condition, for both men and women.

## Introduction

Cardiovascular diseases stand out as the main cause of death^1 - 3^in the world. Latin American countries have high mortality rates from^2^cardiovascular diseases, with Brazil being the country with the highest rates. Arterial hypertension (AH), in turn, is the most common among cardiovascular diseases, and the main risk factor for cerebrovascular diseases.^3 , 4^

AH often develops due to a complex interaction of risk factors associated with lifestyle, genetic predisposition, and aging. This condition affects all socioeconomic groups of both sexes, but has the highest rates among men, especially in low- and middle-income nations.^4^

The *Sociedade Brasileira de Cardiologia* points out in its latest AH guideline that consistent physical activity (PA) plays a crucial role in preventing AH, in addition to contributing to reducing blood pressure (BP) in people who already have this condition. This beneficial effect can be attributed to direct changes in hemodynamic aspects and indirect modifications that occur through nutritional, metabolic, and behavioral adaptations.^1 , 2 , 4^

Studies developed with data from ELSA-Brasil^5 , 6^indicate an inverse association between leisure time physical activity (LTPA) and AH, as well as cardiovascular events, but were carried out cross-sectionally.

From this scenario, the present study can add knowledge to the topic, mainly from the perspective of a better understanding of possible changes in LTPA patterns during monitoring of the studied population and the incidence of AH. Furthermore, it will be able to foment the formulation of public policies that promote and encourage the practice of LTPA by an increasingly larger number of people.

Therefore, this study aims to investigate, longitudinally, the association between LTPA and the incidence of hypertension in ELSA-Brasil participants.

## Methods

### Population and sample

ELSA-Brasil is a cohort study with 15,105 active and retired public servants, aged 35 to 74, from six higher education institutions. The main objective is to investigate the incidence and progression of diabetes and cardiovascular diseases, including AH, and their associated factors, the methodological details of which have been previously described.^7 , 8^For the present study, participants from the baseline (2008-2010) were selected, followed by the 1^st^segment (2012-2014).

To compose the sample for this study, exclusively individuals with normal BP (normotensives) were selected, excluding anyone with a previous diagnosis of hypertension (hypertensives) or who was using medication to treat AH. All participants answered the questionnaires about PA and provided complete data regarding the variables involved in the analysis, totaling 8968 participants, 4,916 women and 4,052 men.

ELSA-Brasil was approved by the National Research Ethics Commission (NREC) and by all Research Ethics Committees of the six research centers involved. All participants signed the free and informed consent form (FICF), ensuring confidentiality and data confidentiality.

### Data production

The data were collected by a team of interviewers and assessors trained and certified by a quality control committee,^7^qualified to execute the study protocol in any ELSA-Brasil Research Center. Face-to-face interviews were carried out when applying blocks of questionnaires to obtain information on age, race/color, education, family income, smoking, and salt consumption. In addition, information was collected on anthropometric measurements of weight, height, and waist circumference. Body weight was obtained in the morning, after 8 to 12 hours of fasting, and with the participant without shoes and wearing light clothing. A Toledo^®^electronic scale was used, with a capacity of up to 200kg. To measure height, a SECA^®^stadiometer was used, with the participant positioned standing up and strictly following the Frankfurt plane. Obesity was identified using the body mass index (BMI), applying the equation BMI = weight (kg)/height (m)^2^.

### Physical activity assessment

To identify and quantify PA, the LTPA module of the International Physical Activity Questionnaire (IPAQ), long version, was used, which consists of questions relating to the frequency and duration of physical activities (walking, moderate PA, and vigorous PA) developed in the work, displacement, domestic activities and leisure time.^9^PA was measured in minutes/week by multiplying the weekly frequency by the duration of each of the activities performed. For this study, the LTPA was used, with the following categorization: 0 = physically inactive (less than 10 minutes of any PA per week); 1 = little active (between 10 minutes and less than 150 minutes of moderate/walking per week, or between 10 minutes and less than 60 minutes of vigorous activity per week, or between 10 minutes and less than 150 minutes per week of any combination of moderate and vigorous walking); 2 = physically active (at least 150 minutes of walking/moderate activity per week, or at least 60 minutes of vigorous activity per week, or at least 150 minutes per week of any combination of moderate and vigorous PA); 3 = very active (at least 150 minutes of vigorous activity per week, or at least 60 minutes of vigorous activity per week plus 150 minutes per week of any combination of moderate and vigorous PA).

Analyses with LTPA were categorized into two groups, those classified as little active were considered as insufficiently active, and those classified as active as sufficiently active. For LTPA analyses, four categories were considered: inactive, slightly active, active, and very active.

### Assessment of arterial hypertension

AH was defined as SBP ≥ 140 mm/Hg and DBP ≥ 90 mm/Hg.BP was measured with a digital sphygmomanometer (Omron HEM-705CP). BP measurements were taken after five minutes of rest, with the participant with an empty bladder, sitting upright, with their back relaxed and supported on the backrest, feet supported, legs uncrossed, and left arm resting at heart level. Three measurements were taken at one-minute intervals. The BP used in the present study was established by the arithmetic mean of the second and third measurements.

### Covariates

For the covariates, the following categorization was adopted: Sex: man and woman; For race/color: black, brown, white, Asian, indigenous; For age: between 34 and 50 years, between 51 and 60 years and >60 years; for education, four categories were established: incomplete elementary school, completed elementary school, completed high school and completed higher education/postgraduate studies. Family income was categorized as up to 2 minimum wages, from 2 to 8 minimum wages, from 8 to 18 minimum wages, and above 18 minimum wages. Current smoking was categorized as no and yes; For BMI: < 30 kg/m², = ≥ 30 kg/m²; for salt consumption/day: < 5g and = ≥ 5g.

### Data analysis

Descriptive measures (proportions) were calculated for all categorized variables. Analyses were stratified by sex a priori and compared using the Chi-square test. The associations were analyzed using Poisson regression, estimating the Relative Risk (RR) with a 95% confidence interval. The following variables were considered as potential confounders or effect modifiers: age, BMI, smoking, family income, salt consumption, race/color, and education.

Verification of effect modification was carried out through stratification with the observation of strato-specific punctual measurements and their intervals and confidence. When the point measurement of a factor, in a specific stratum, was not contained in the confidence interval of the other factor in the same stratum, this indicated effect modification. The analysis of possible confounding variables was carried out using the backward procedure, using Poisson regression. The analysis starts with the full model, followed by one-by-one removal of potential confounders. When a change equal to or greater than 10% is observed in the specific association between LTPA and AH, the variable is considered confounding.^10^Although no effect-modifying and/or confounding variables were identified in the modeling process, adjustment by age was chosen, since, theoretically, both LTPA and AH are related to age.

Longitudinal analyses were carried out, calculating the RR between LTPA and AH using the insufficiently active category as a reference for the analysis with two categories, and the inactive group as a reference for the analysis with four categories.

The Mantel-Haenszel test was used to test the homogeneity of RR values between the strata of each variable. The level of significance adopted in the statistical analysis was 5%. The confidence interval was set at 95%. The statistical program STATA® version 17.0 was used.

## Results

A total of 4,052 men and 4,916 women were included in the analysis. The characteristics of the sample are presented in Table 1 . The incidence of AH in the first segment was 16.9%, 12.8% among women, and 21.9% among men, however, the majority of participants were female (55%), among them 37.8% were aged between 34-50 years. It is also observed that women have a higher family income and are more active in their leisure time, while men are more smokers, more hypertensive, and have a higher proportion of very active participants in their leisure time. It is also observed that there are no statistically significant differences between men and women concerning age. About 16% of both sexes were sufficiently active at baseline and became insufficiently active, and approximately 13% of the insufficiently active became sufficiently active. Around 8% remained very physically active at both follow-up times.

**Table 1:** Distribution of participants according to selected characteristics and gender. ELSA-Brasil 2008-2014

		Men	Women	p-value
Age (years) - n (%)		(4.052)	(4.916)	
	34-50	1642 (46.71)	1859 (53.10)	
	51-60	1427 (44.32)	1793 (55.68)	
	> 60	983 (43.75)	1264 (56.38)	0.030
Race/self-referred color - n (%)				
	Black	479 (41.73)	680 (58.67)	
	Brown	1229 (49.62)	1248 (50.38)	
	White	2171 (44.05)	2758 (55.95)	
	Asian	68 (30.49)	155 (69.51)	
	Indigenous	50 (60.24)	33 (39.76)	0.00
Family income (minimum wage) - n (%)				
	Until 2	40 (42.55)	54 (57.45)	
	From 2 to 8	1559 (47.48)	1715 (52.22)	
	From 2 to 18	1385 (40.82)	2008 (59.18)	
	Above 18	1041 (48.13)	1922 (51.87)	0.00
Education - n (%)				
	Incomplete elementary school	253 (71.67)	100 (28.33)	
	Complete elementary school	303 (60.84)	195 (39.16)	
	Complete high school	1323 (45.06)	1613 (54.94)	
	Complete higher education/Postgraduate	2173 (41.94)	1756 (49.84)	0.00
Current smoking - n (%)				
	No	2276 (41.90)	3156 (58.10)	
	Yes	1767 (50.71)	1756 (49.84)	0.00
BMI - n (%)				
	< 30 kg/m²	3354 (46.81)	3811 (53.19)	
	≥ 30 kg/m²	698 (38.71)	1015 (61.19)	0.00
Salt consumption - n (%)				
	< 5g	203 (22.61)	695 (77.39)	
	≥ 5g	3849 (47.70)	4121 (52.30)	0.00
Changes in LTPA (between study stages - n (%)				
	Insufficient LTPA – Insufficient LPTA	1296 (39.12)	2017 (60.88)	
	Enough LTPA – Enough LPTA	217 (45.30)	262 (54.70)	
	Enough LTPA – Insufficient LPTA	637 (44.73)	787 (55.27)	
	Insufficient LTPA – Sufficient LPTA	514 (45.65)	612 (54.35)	0.00
LPTA Levels - n (%)				
	Inactive	888 (38.81)	1400 (61.19)	
	Less active	217 (45.30)	262 (54.70)	
	Active	411 (46.97)	464 (53.03)	
	Very active	461 (63.24)	268 (36.24)	0.00
Arterial hypertension - n (%)				
	No	3148 (42.45)	4267 (57.55)	
	Yes	887 (58.43)	631 (41.57)	0.00

* Values for men and women were obtained using the chi-square test. The sums of the strata will not always be equal, due to the loss of information on some variables. BMI: body mass index; LTPA: leisure time physical activity.

As seen in Table 2 , there were no significant statistical associations between changes in LTPA and AH among participants when using the two-category classification.

**Table 2:** Association between changes in LPTA between two segments and incidence of AH in ELSA-Brasil participants (male and female)

Changes in LTPA	MenRR (CI 95%)	WomenRR (CI 95%)
Insufficient LTPA – Insufficient LTPA	1.00	1.00
Sufficient LTPA – Sufficient LTPA	1.00 (0.75-1.35)	1.06 (0.76-1.48)
Insufficient LTPA – Sufficient LTPA	0.95 (0.78-1.16)	0.87 (0.69-1.10)
Sufficient LTPA – insufficient LTPA	0.81 (0.64-1.02)	0.80 (0.62-1.04)

Adjusted by age. RR: relative risk; LTPA: leisure time physical activity.

Table 3 presents the analysis using the level of LTPA in four categories, which indicated a statistical association between LTPA and AH in very active participants: risk of AH reduced by 35% among men RR = 0.65 (95% CI 0.50 −0.86) and risk of AH reduced by 66% among women RR = 0.34 (95% CI 0.20- 0.58). The Central Illustration summarizes the main information of the manuscript.

**Table 3:** Association between LPTA levels and incidence of AH in ELSA-Brasil participants (male and female)

Níveis de ATFL	Men RR (CI 95%)	Women RR (CI 95%)
Inactive – Inactive	1.00	1.00
Less Active – Less active	1.03 (0.76-1.40)	0.99 (0.71-1.39)
Active – Active	0.83 (0.64-1.07)	0.77 (0.58-1.03)
Very Active – Very active	0.65 (0.50-0.86)	0.34 (0.20-0.58)

Adjusted by age. RR: relative risk; LTPA: leisure time physical activity.

## Discussion

This study analyzed associations between changes in LTPA during the follow-up period and incidence of AH. LTPA, particularly in the very physically active group, was associated with a lower risk of developing hypertension over time.

After statistical analysis, considering participants classified into two categories, the first results did not reveal significant statistical associations between LTPA and AH. On the other hand, among participants classified into four categories, those considered as "very active" physically, both men and women who maintained high levels of LTPA throughout the study had a significantly lower risk of developing AH. Furthermore, women had an even more pronounced reduction in risk compared to men.

The significantly lower risk of developing AH in very active individuals can be explained by several factors. First, the intensity and regularity of PA play a crucial role in reducing BP.^11^Very active people generally engage in more intense and frequent PA, which contributes to improved cardiovascular health and the body's ability to regulate BP more effectively.^11 , 12^

Furthermore, intense PA may lead to beneficial changes in other risk factors associated with hypertension, such as weight control, insulin sensitivity, and vascular function. Very active people spend more energy while maintaining a lower percentage of body fat, which may be linked to a lower incidence of AH.^2 , 3 , 11^

It is also important to consider that intense PA is generally associated with healthier lifestyles in terms of diet and behavioral habits. Very active individuals are often more conscious of their health, which can lead to better food choices and reduced risk factors such as salt consumption.^3 , 11 , 12^

However, this does not mean that moderate PA is ineffective in preventing AH, as suggested by a study,^13^which for the prevention of conditions such as hypertension, cardiovascular events, and even diabetes, performing LTPA with moderate to vigorous intensity has already been shown to be efficient.

From this perspective, a previous cross-sectional study with data from the ELSA-Brasil baseline showed an inverse association between LTPA and AH in individuals who practiced LTPA with moderate intensity.^5^Along the same lines, another study pointed out that PA in the domestic domain, of low and moderate intensity was associated with a lower risk for AH.^14^

On the other hand, a study also using data from ELSA-Brasil verified the association between LTPA and PA during commuting with cardiovascular risk scores, showing that only LTPA with higher duration and intensity was inversely associated with AH and cardiovascular events.^6^

Our results corroborate other longitudinal studies, meta-analyses, and even clinical trials,^11 , 15 , 16^which demonstrate that the reduction in risk for AH is progressively linked to the volume and intensity of PA practiced and that the greater the volume and/or intensity of PA, the lower the risk of developing AH.

It is essential to emphasize that our research has some notable characteristics, despite the population studied consisting of a cohort of volunteer public servants. Firstly, the cohort is made up of a substantial number of participants from six Brazilian capitals. This regional diversity is a positive aspect of the study, as it can provide insights into AH in different geographic and cultural contexts in Brazil. This, in turn, contributes to the generalization of results and a more comprehensive understanding of the factors that affect hypertension in the Brazilian population.

Besides that, choosing volunteer public servants as a study group may have advantages, as this group may have better access to healthcare, health information, and awareness about the importance of monitoring and controlling BP. Therefore, research can provide valuable information about prevention and awareness strategies that can be applied in a broader context.

Although the study population may not be fully representative of the general population, its unique characteristics may provide valuable insights into AH and serve as a basis for future research and interventions aimed at cardiovascular health in Brazil.

An unfavorable aspect was the use of questionnaires, as answers can be inaccurate due to dependence on memory. We suggest adopting the accelerometer for more objective measurements of PA in future studies, as is already being done in ELSA-Brasil.

## Conclusion

According to the results of this study, LTPA was associated with a reduced risk of AH in participants who maintained higher levels of LTPA at both moments analyzed. The more physically active you are, the lower your risk of developing AH, regardless of gender. This highlights the crucial role of PA in reducing the risk of hypertension, especially when practiced consistently. These findings emphasize the importance of promoting an active lifestyle as a preventive measure against AH.

Future research should continue to explore the association between more vigorous LTPA and AH, including the use of accelerometry, to provide stronger guidelines for public policies to promote PA and health.
